# Association of Diabetes Mellitus and Obesity with Early Complications After Surgical Tracheostomy in Critically Ill ICU Patients: A Retrospective Observational Cohort Study

**DOI:** 10.3390/life16081251

**Published:** 2026-07-29

**Authors:** Lukas S. Fiedler, Tobias Meyer, Fabian Burk, Fynn Roters

**Affiliations:** 1 ENT and Head and Neck Surgery, Plastic Operations, SLK Kliniken Heilbronn, 74078 Heilbronn, Germany; 2 Faculty of Medicine, Medical University of Heidelberg, 74078 Heidelberg, Germany; 3 ENT and Head and Neck Surgery, Klinikum Mutterhaus der Borromäerinnen Trier, 54290 Trier, Germany; 4 Department of Otorhinolaryngology, Plastic Surgery, Klinikum Stuttgart, 70174 Stuttgart, Germany; 5 Department of Otorhinolaryngology, University Medical Center Hamburg-Eppendorf, 20251 Hamburg, Germany

**Keywords:** surgical tracheostomy, diabetes mellitus, obesity, wound infection, intensive care unit (ICU)

## Abstract

Background: Obesity and diabetes mellitus (DM) are established risk factors for impaired wound healing, but their associations with early postoperative complications following surgical tracheostomy in critically ill patients remain insufficiently characterized. This study evaluated the associations of obesity and DM with clinically documented early peristomal wound infection following bedside surgical tracheostomy using either the Visor or Björk technique. Methods: This retrospective single-centre cohort study included 92 ICU patients who underwent bedside surgical tracheostomy between 2022 and 2023. Postoperative complications were retrospectively assessed from clinical documentation at postoperative days 5–7 and 10–14. Documentation at days 5–7 was available for all 92 patients, whereas documentation at days 10–14 was available for 78 patients. The analyses therefore evaluated complications documented during the available postoperative follow-up period of up to 14 days rather than complete 14-day cumulative incidence. Associations of obesity and DM with clinically documented peristomal wound infection were assessed using exploratory univariable analyses. Results: Clinically documented peristomal wound infection occurred in 18 of 92 patients (19.6%) during the available postoperative follow-up period. Infection was documented in 10 of 28 patients with obesity (35.7%) compared with 8 of 64 patients without obesity (12.5%; crude OR 3.89, 95% CI 1.33–11.35; Fisher’s exact *p* = 0.020). Infection was also more frequent in patients with DM (12/31, 38.7%) than in those without DM (6/61, 9.8%; crude OR 5.79, 95% CI 1.91–17.57; Fisher’s exact *p* = 0.002). Postoperative haemorrhage was numerically more frequent in patients with obesity (14.3% vs. 6.3%), but this association was not statistically significant (crude OR 2.50, 95% CI 0.58–10.78; Fisher’s exact *p* = 0.242). Conclusions: Obesity and DM were associated with higher crude odds of clinically documented early peristomal wound infection following bedside surgical tracheostomy. Given the exploratory, unadjusted analyses and incomplete follow-up through days 10–14, these findings should be considered hypothesis-generating and require confirmation in larger prospective cohorts.

## 1. Introduction

Tracheostomy is one of the oldest surgical procedures, with depictions dating back to 2950–2800 BC [1]. In modern intensive care medicine, it represents an essential intervention to secure the airway in patients requiring prolonged or acute mechanical ventilation [2]. Terminology remains heterogeneous: while tracheotomy describes the opening of the trachea, tracheostomy refers to the creation of a mucocutaneous anastomosis forming an epithelialized stoma between the trachea and the skin [2,3]. Various surgical techniques have been developed, differing mainly in incision design, stoma configuration, and preservation of tracheal structures [2].

Surgical tracheostomy is indicated in patients requiring prolonged ventilation or difficult weaning and may also be performed in cases of airway obstruction and specific airway pathologies [2,4,5,6]. In patients with an unfavourable anatomical condition including obesity, goitre, or distorted cervical anatomy, percutaneous dilatational techniques may be contraindicated, and surgical approaches are preferred [2]. Among the established techniques, the Björk tracheostomy (BTS), first described in 1955 [7], and the more recent Visor-tracheostomy (VTS), introduced in 2004 [8], are widely used. The VTS offers a cartilage-sparing, blood-supply-preserving modification that may promote improved wound healing and reduce subglottic stenosis [3,9,10]. Both procedures are feasible under operating room and bedside conditions in the intensive care unit (ICU).

Despite its routine use, surgical tracheostomy is associated with a range of early and late complications. Early complications include haemorrhage, subcutaneous emphysema, tube dislocation, and peristomal wound infection, the latter representing a clinically relevant cause of morbidity and, in severe cases, potentially progressing to necrotizing fasciitis or mediastinitis [11,12,13]. Although surgical technique may influence local tissue trauma and wound healing, patient-related factors may also contribute substantially to postoperative outcomes.

Metabolic comorbidities, particularly obesity and diabetes mellitus (DM), are clinically relevant factors in surgical wound healing. Obesity has been associated with impaired tissue oxygenation, altered inflammatory responses, and an increased susceptibility to nosocomial and surgical infections [14,15]. Diabetes mellitus may impair tissue repair through hyperglycaemia-associated microvascular dysfunction and altered immune responses and has been associated with surgical-site infection across different surgical populations [16]. These mechanisms may be particularly relevant in surgical tracheostomy. In patients with obesity, increased pretracheal soft-tissue thickness may complicate surgical exposure and potentially affect local wound conditions, while diabetes may further compromise tissue repair and host defence. However, despite evidence from other surgical populations, the specific associations of obesity and diabetes mellitus with early peristomal wound complications following bedside surgical tracheostomy remain insufficiently characterized.

The present retrospective cohort study investigated the associations of obesity and diabetes mellitus with clinically documented early peristomal wound infection following bedside surgical tracheostomy performed using either the Visor or Björk technique. Postoperative haemorrhage and other documented early complications were assessed as secondary outcomes.

## 2. Materials and Methods

### 2.1. Ethics Statement

This retrospective study was conducted in accordance with the principles of the Declaration of Helsinki. Ethical approval was obtained from the Ethics Committee of the Medical Association of Hamburg (reference number 2024-101420-BO-ff; approval date: 29 January 2025). The ethics approval covered the retrospective use and analysis of routinely collected clinical data. According to § 12 of the Hamburg Hospital Act, individual informed consent was not required for this retrospective study.

### 2.2. Study Design and Setting

This retrospective, single-centre observational cohort study was conducted at the Department of Otorhinolaryngology, University Medical Center Hamburg-Eppendorf, Germany. Consecutive adult patients admitted to the intensive care unit (ICU) who underwent bedside surgical tracheostomy between January 2022 and December 2023 were screened for eligibility. Clinical data were retrospectively extracted from the institutional electronic medical record system (Soarian Clinicals^®^, Cerner Corporation, North Kansas City, MO, USA) and analysed in pseudonymized form. The study was designed to explore the associations of obesity and diabetes mellitus with clinically documented early peristomal wound complications following bedside surgical tracheostomy.

### 2.3. Study Population

Consecutive adult ICU patients undergoing bedside surgical tracheostomy by the otorhinolaryngology team during the study period were screened for inclusion. Patients were eligible if they were ≥18 years of age and underwent surgical tracheostomy in the ICU between January 2022 and December 2023. A total of 101 potentially eligible patient records were identified. Before the first postoperative assessment at days 5–7, nine patients were not available for analysis because of decannulation (*n* = 1), death (*n* = 3), interhospital transfer (*n* = 2), or insufficient postoperative documentation (*n* = 3), resulting in an analysis cohort of 92 patients.

Postoperative documentation at days 5–7 was available for all 92 patients. Documentation for the second assessment period at days 10–14 was available for 78 patients. The reduction in available follow-up was attributable to decannulation (*n* = 4), death (*n* = 5), or interhospital transfer (*n* = 5). Consequently, analyses based on the full cohort represent complications documented during the available postoperative follow-up period of up to 14 days rather than complete 14-day cumulative incidence.

**Figure 1:**
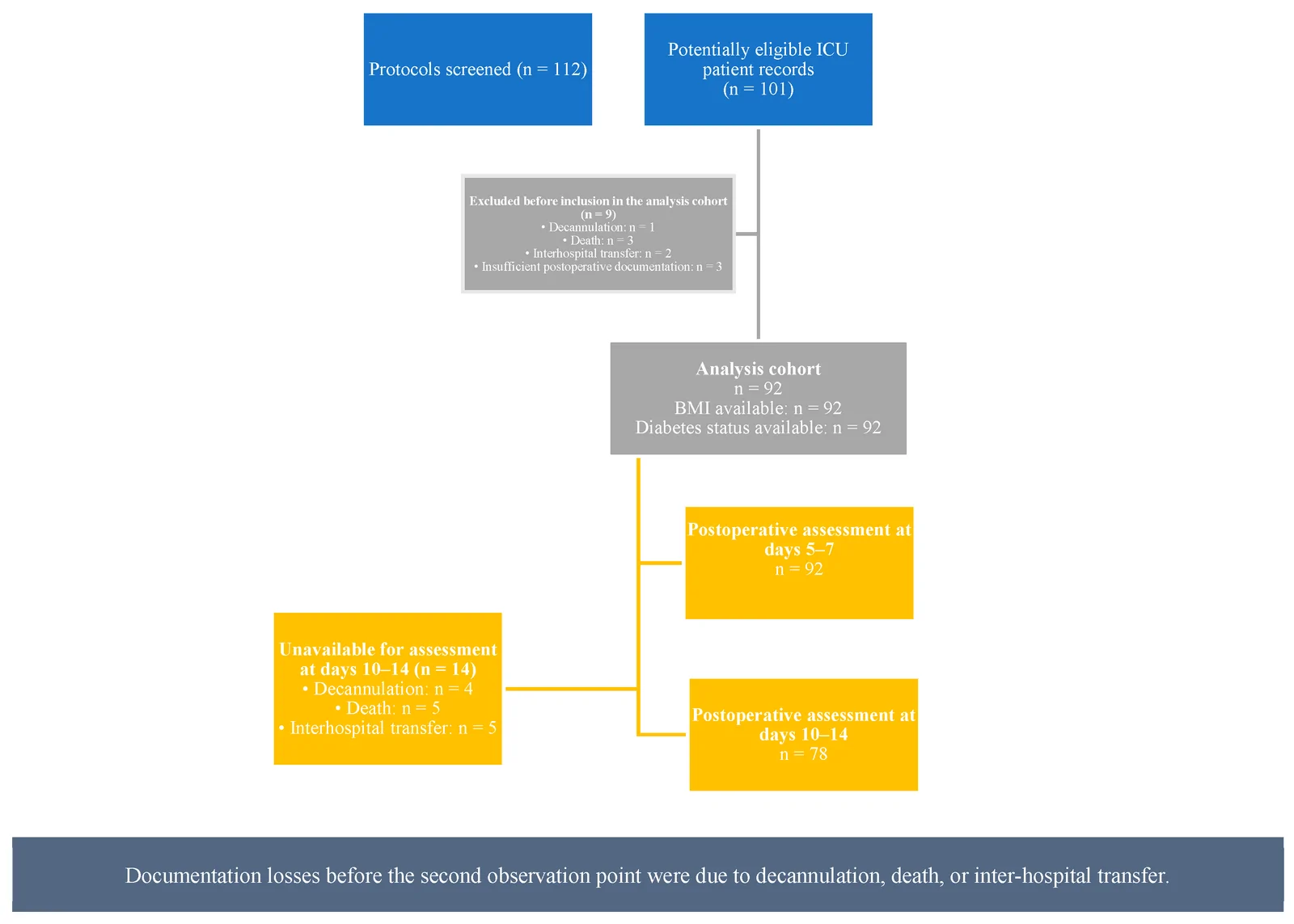
STROBE-style flow diagram of patient selection and availability of postoperative follow-up data at days 5–7 and days 10–14.

Similarly, the timing of individual clinically documented peristomal wound infections could not be reliably reconstructed between the two predefined assessment periods from the available historical participant-level dataset. Therefore, neither a complete-case sensitivity analysis restricted to the 78 patients nor a reliable comparison of infection occurrence between days 5–7 and days 10–14 was performed. Patient selection and follow-up availability are summarized in Figure 1. Indications for surgical tracheostomy were determined according to routine institutional clinical practice and included prolonged mechanical ventilation and difficult weaning from ventilatory support. Individual indications were not systematically available for the present retrospective analysis.

### 2.4. Tracheostomy Techniques

Two standardized bedside surgical tracheostomy techniques were used: Björk tracheostomy (BTS) and Visor tracheostomy (VTS). Both procedures were performed under general anaesthesia and standardized aseptic conditions in the ICU by experienced otorhinolaryngology surgeons in collaboration with an anaesthesiologist.

In BTS, an inferiorly based tracheal wall flap was created and sutured to the skin to establish a stable mucocutaneous tract. VTS was performed as a cartilage-preserving technique using an intercartilaginous horizontal tracheal incision, followed by fixation of the tracheal opening to the skin. The surgical techniques have been described in detail previously [3].

### 2.5. Data Collection

Clinical and demographic data were extracted retrospectively from the electronic medical records. Variables available for the present analysis included age, sex, body mass index (BMI), documented diabetes mellitus, tracheostomy technique, and postoperative complications. Follow-up information was obtained from routine postoperative clinical documentation.

Diabetes mellitus was defined as a documented pre-existing diagnosis in the medical record. Information on diabetes subtype, HbA1c, perioperative blood glucose levels, antidiabetic treatment, and duration of diabetes was not consistently available. Similarly, several potentially relevant clinical confounders, including standardized illness-severity scores, smoking status, nutritional status, corticosteroid or immunosuppressive treatment, perioperative antibiotic exposure, and duration of mechanical ventilation, were not systematically available for analysis and the available historical dataset did not permit reliable patient-level reconstruction of the timing of individual wound-infection events between the two predefined assessment periods.

### 2.6. Exposure Variables

The primary exposure variables were obesity and diabetes mellitus. Obesity was defined as BMI ≥ 30 kg/m^2^ according to the World Health Organization classification. Patients were therefore categorized as having obesity (BMI ≥ 30 kg/m^2^) or not having obesity (BMI < 30 kg/m^2^). Diabetes mellitus was analysed as a binary variable based on a documented pre-existing diagnosis in the medical record.

### 2.7. Outcome Measures

The primary outcome was clinically documented peristomal wound infection during the available postoperative follow-up period of up to 14 days. Wound infection was identified retrospectively from routine clinical documentation and included documented peristomal erythema and/or swelling, putrid wound secretion, or an explicit clinical diagnosis of peristomal infection. Wound necrosis was recorded separately as a subsequent local wound complication.

Because these findings were derived from routine retrospective documentation, erythema and swelling could not always be reliably distinguished from non-infectious postoperative inflammatory changes. The outcome should therefore be interpreted as clinically documented peristomal wound infection rather than as a standardized surgical-site infection endpoint.

Secondary outcomes included postoperative haemorrhage, subcutaneous emphysema, wound dehiscence, false passage, and surgical revision. Postoperative outcomes were assessed from routine documentation during two predefined assessment periods at postoperative days 5–7 and days 10–14. Postoperative haemorrhage was defined as clinically documented bleeding considered temporally and clinically related to the tracheostomy procedure or associated airway manipulation during the perioperative period. Bleeding events requiring surgical revision were additionally classified according to their documented source and management.

### 2.8. Statistical Analysis

Statistical analyses were performed using R (version 4.5.0; R Foundation for Statistical Computing, Vienna, Austria) and IBM SPSS Statistics for macOS (version 29.0.2.0; IBM Corp., Armonk, NY, USA). Continuous variables are presented as mean ± standard deviation (SD) or median [interquartile range (IQR)], as appropriate, and categorical variables as absolute and relative frequencies.

Exploratory univariable analyses were performed to evaluate the crude associations of obesity and diabetes mellitus with clinically documented peristomal wound infection. Obesity was defined as BMI ≥ 30 kg/m^2^. Categorical variables were compared using Fisher’s exact test because of the limited number of outcome events and small expected cell counts. Effect estimates are reported as crude odds ratios (ORs) with corresponding 95% confidence intervals (CIs).

Multivariable modelling was not performed because of the limited number of wound-infection events relative to the number of clinically relevant potential confounders. Consequently, the reported effect estimates represent crude associations and should not be interpreted as independent effects. All statistical tests were two-sided, and *p* < 0.05 was considered statistically significant. Given the exploratory nature of the analyses, no adjustment for multiple testing was performed.

## 3. Results

### 3.1. Cohort Characteristics and Follow-Up

A total of 92 patients were included in the analysis cohort. Of these, 68 (73.9%) underwent Visor tracheostomy (VTS) and 24 (26.1%) underwent Björk tracheostomy (BTS). The mean age was 59.2 ± 13.9 years, and 62 patients (67.4%) were male. Obesity (BMI ≥ 30 kg/m^2^) was present in 28 patients (30.4%), and 31 patients (33.7%) had documented diabetes mellitus. Baseline characteristics are summarized in Table 1.

Postoperative assessment at days 5–7 was documented for all 92 patients. Documentation for the second assessment period at days 10–14 was available for 78 patients. Fourteen patients were no longer available for the second assessment because of decannulation (*n* = 4), death (*n* = 5), or interhospital transfer (*n* = 5).

### 3.2. Postoperative Complications

During the available postoperative follow-up period of up to 14 days, at least one postoperative complication was documented in 28 of 92 patients (30.4%). Complications were documented in 20 of 68 patients undergoing VTS (29.4%) and 8 of 24 patients undergoing BTS (33.3%), with no statistically significant difference between techniques (Fisher’s exact *p* = 0.798). The most frequently documented complications were peristomal wound infection and postoperative haemorrhage (Table 2). Among the 92 patients included in the analysis cohort, five all-cause deaths occurred before the second postoperative assessment. None of these deaths was documented as directly attributable to the tracheostomy procedure.

### 3.3. Wound Infections

A total of 18 patients (19.6%) had a clinically documented peristomal wound infection during the available postoperative follow-up period. Among these patients, 12 had documented peristomal erythema and/or swelling and 6 had putrid wound secretion. Two patients with a previously documented wound infection subsequently developed local wound necrosis; these events were therefore considered progression of an existing wound complication rather than additional infection events.

### 3.4. Obesity and Clinically Documented Peristomal Wound Infection

Clinically documented peristomal wound infection was observed in 10 of 28 patients with obesity (35.7%) and in 8 of 64 patients without obesity (12.5%). In exploratory univariable analysis, obesity was associated with higher crude odds of documented wound infection (crude OR 3.89, 95% CI 1.33–11.35; Fisher’s exact *p* = 0.020) (Table 3).

### 3.5. Obesity and Postoperative Haemorrhage

Postoperative haemorrhage was documented in 4 of 28 patients with obesity (14.3%) and in 4 of 64 patients without obesity (6.3%). Although haemorrhage was numerically more frequent among patients with obesity, the crude association was not statistically significant (crude OR 2.50, 95% CI 0.58–10.78; Fisher’s exact *p* = 0.242) (Table 4).

Eight postoperative haemorrhagic complications were documented. Two patients required surgical revision for bleeding-related complications. In one case, bleeding originated from the pharynx following an intubation-related injury in the context of airway management during tracheostomy. In the second case, bleeding originated from the tracheostomy field and was controlled surgically by coagulation. Both events were classified as procedure-associated postoperative haemorrhagic complications.

### 3.6. Diabetes Mellitus and Clinically Documented Peristomal Wound Infection

Clinically documented peristomal wound infection was observed in 12 of 31 patients with diabetes mellitus (38.7%) compared with 6 of 61 patients without diabetes mellitus (9.8%). In exploratory univariable analysis, diabetes mellitus was associated with higher crude odds of documented wound infection (crude OR 5.79, 95% CI 1.91–17.57; Fisher’s exact *p* = 0.002) (Table 5).

### 3.7. Association Between Obesity and Diabetes Mellitus

Diabetes mellitus was documented in 16 of 28 patients with obesity (57.1%) compared with 15 of 64 patients without obesity (23.4%). Obesity was associated with higher crude odds of prevalent diabetes mellitus (crude OR 4.36, 95% CI 1.70–11.19; Fisher’s exact *p* = 0.003).

## 4. Discussion

This retrospective observational study identified crude associations between obesity, diabetes mellitus, and clinically documented peristomal wound infection following bedside surgical tracheostomy in critically ill patients. Wound infection was documented more frequently in patients with obesity than in those without obesity (35.7% vs. 12.5%) and in patients with diabetes mellitus than in those without diabetes (38.7% vs. 9.8%). In contrast, postoperative haemorrhage was numerically more frequent in patients with obesity, but the association was not statistically significant. Importantly, obesity and diabetes mellitus were themselves strongly associated within the cohort. Given the exploratory univariable analyses and the limited number of outcome events, these findings should be interpreted as crude associations rather than evidence of independent risk effects.

Obesity is a recognized contributor to impaired wound healing and postoperative morbidity across surgical disciplines [14]. Potential mechanisms include chronic low-grade inflammation, altered immune responses, impaired tissue oxygenation, and reduced microvascular perfusion [15]. These mechanisms may be particularly relevant in surgical tracheostomy, where increased pretracheal soft-tissue thickness and greater skin-to-trachea distance can complicate surgical exposure and local wound management [2]. In the present cohort, clinically documented peristomal wound infection was more frequent among patients with obesity. However, because the analysis was unadjusted, this association cannot be attributed to obesity independently of diabetes mellitus or other clinical characteristics.

Diabetes mellitus was likewise associated with a higher frequency of clinically documented peristomal wound infection in our cohort. This observation is biologically plausible and consistent with evidence from other surgical settings. Diabetes may impair wound healing through microvascular dysfunction, altered leukocyte activity, impaired epithelialization, and reduced host defence. In head and neck surgery, diabetes has previously been associated with increased rates of surgical-site infection [16], while prospective tracheostomy studies have identified diabetes among factors associated with postoperative complications and hospital readmission [17]. Nevertheless, the present data do not establish diabetes mellitus as an independent predictor of wound infection because data on glycaemic control and several relevant clinical confounders were unavailable and no adjusted analysis was performed.

A particularly relevant finding was the substantial overlap between obesity and diabetes mellitus. Diabetes was present in 57.1% of patients with obesity compared with 23.4% of those without obesity. Consequently, the separate crude associations observed for obesity and diabetes mellitus should not be interpreted as independent effects. Both exposures may represent overlapping components of a broader metabolic and clinical risk profile. The limited number of wound-infection events precluded robust multivariable adjustment for these and other potentially relevant confounders.

Postoperative haemorrhage was numerically more frequent among patients with obesity (14.3% vs. 6.3%), although this difference was not statistically significant and the confidence interval was wide. Anatomical factors associated with obesity, including increased pretracheal tissue thickness and greater surgical exposure requirements, may plausibly influence procedural complexity [2,15]. However, the small number of haemorrhagic events in the present cohort does not permit conclusions regarding an association between obesity and postoperative bleeding.

From a clinical perspective, the observed associations support awareness of metabolic comorbidities when evaluating patients undergoing bedside surgical tracheostomy. However, the present study does not demonstrate that specific interventions, such as intensified glycaemic management or individualized antibiotic prophylaxis, reduce postoperative wound complications. Whether targeted perioperative strategies improve outcomes in metabolically vulnerable patients should be evaluated prospectively.

Several limitations should be considered when interpreting these findings. First, the retrospective single-centre design and relatively small sample size limit statistical power and generalisability. Only 18 clinically documented peristomal wound infections occurred, precluding robust multivariable adjustment for the numerous potentially relevant confounders. The reported odds ratios therefore represent crude associations and should not be interpreted as independent effects.

Second, the definition of peristomal wound infection was based on routine retrospective clinical documentation rather than a prospectively applied standardized surgical-site infection definition. Erythema and swelling may overlap with postoperative inflammatory changes, introducing potential outcome misclassification. Of the 18 documented infections, 12 were characterized by erythema and/or swelling and six by putrid wound secretion; two patients subsequently developed wound necrosis. Microbiological sampling, infection-specific antimicrobial treatment, and other infection-related interventions could not be consistently reconstructed.

Third, postoperative follow-up was incomplete. While all 92 patients had documented assessment at postoperative days 5–7, documentation at days 10–14 was available for 78 patients. Because patient-level membership of this later follow-up subgroup could not be reliably reconstructed according to obesity or diabetes status from the available historical dataset, differential attrition according to metabolic exposure could not be assessed and a complete-case sensitivity analysis was not feasible. Consequently, the reported outcomes represent complications documented during the available follow-up period of up to 14 days rather than complete 14-day cumulative incidence.

Finally, several clinically relevant potential confounders were not systematically available, including illness severity, smoking and nutritional status, corticosteroid or immunosuppressive therapy, perioperative antibiotic exposure, glycaemic control, duration of mechanical ventilation, and other treatment-related factors. Residual and unmeasured confounding therefore remains substantial.

Taken together, the present findings suggest crude associations of obesity and diabetes mellitus with clinically documented early peristomal wound infection after bedside surgical tracheostomy. The findings are biologically plausible and consistent with the broader surgical literature, but their independence and magnitude cannot be established from the present dataset. Larger prospective multicentre studies using standardized infection definitions, complete follow-up, detailed metabolic characterization, and appropriate adjustment for clinical confounders are needed to determine whether obesity and diabetes independently contribute to wound complications after surgical tracheostomy.

## 5. Conclusions

In this retrospective cohort of critically ill patients undergoing bedside surgical tracheostomy, obesity and diabetes mellitus were associated with higher crude odds of clinically documented peristomal wound infection during the available postoperative follow-up period of up to 14 days. In contrast, no statistically significant association was observed between obesity and postoperative haemorrhage.

Despite the limitations inherent to the retrospective design and incomplete follow-up, this study provides clinically relevant data on a scarcely investigated aspect of surgical tracheostomy by specifically examining metabolic patient characteristics in relation to early wound complications. The observed associations, together with the substantial overlap between obesity and diabetes, highlight the potential relevance of metabolic comorbidity in perioperative risk assessment while emphasizing the need for cautious interpretation of individual risk factors. These findings provide a basis for larger prospective studies using standardized outcome definitions, complete follow-up, and multivariable adjustment to determine the independent contribution of metabolic factors to wound complications after surgical tracheostomy.

## Figures and Tables

**Table 1:** Data are presented for the analysis cohort (*n* = 92). BMI and diabetes status were available for all 92 patients; no exposure data were missing.

Characteristic	Overall Cohort (*n* = 92)
Age, years, mean ± SD	59.2 ± 13.9
Male sex, *n* (%)	62 (67.4)
Obesity (BMI ≥ 30 kg/m^2^), *n* (%)	28 (30.4)
Diabetes mellitus, *n* (%)	31 (33.7)
Visor tracheostomy, *n* (%)	68 (73.9)
Björk tracheostomy, *n* (%)	24 (26.1)

**Table 2:** Postoperative complications documented during the available follow-up period of up to 14 days. Individual patients could experience more than one complication.

Complication	*n* (%)
Clinically documented peristomal wound infection	18 (19.6)
Postoperative haemorrhage	8 (8.7)
Subcutaneous emphysema	1 (1.1)
Surgical revision	2 (2.2)
Subsequent wound necrosis *	2 (2.2)
Wound dehiscence	0
False passage	0

* two patients with a previously documented wound infection subsequently developed local wound necrosis.

**Table 3:** Association between obesity and clinically documented peristomal wound infection during the available postoperative follow-up period. Denominator: *n* = 92. Outcomes represent complications documented during the available postoperative follow-up period of up to 14 days. Follow-up documentation at postoperative days 10–14 was available for 78 patients.

BMI Category	Wound Infection (+)	Wound Infection (−)	Total
BMI ≥ 30 kg/m^2^	10 (35.7%)	18 (64.3%)	28
BMI < 30 kg/m^2^	8 (12.5%)	56 (87.5%)	64
Total	18 (19.6%)	74 (80.4%)	92

Crude OR 3.89; 95% CI 1.33–11.35; Fisher’s exact test *p* = 0.020.

**Table 4:** Association between BMI category and postoperative haemorrhage documented during the available follow-up period of up to 14 days. Denominator: *n* = 92. Outcomes represent complications documented during the available postoperative follow-up period of up to 14 days. Follow-up documentation at postoperative days 10–14 was available for 78 patients.

BMI Category	Haemorrhage (+)	Haemorrhage (−)	Total
BMI ≥ 30 kg/m^2^	4 (14.3%)	24 (85.7%)	28
BMI < 30 kg/m^2^	4 (6.3%)	60 (93.8%)	64
Total	8 (8.7%)	84 (91.3%)	92

Crude OR 2.50; 95% CI 0.58–10.78; Fisher’s exact test *p* = 0.242.

**Table 5:** Association between diabetes mellitus and clinically documented peristomal wound infection during the available postoperative follow-up period. Denominator: *n* = 92. Outcomes represent complications documented during the available postoperative follow-up period of up to 14 days. Follow-up documentation at postoperative days 10–14 was available for 78 patients.

Diabetes Status	Wound Infection (+)	Wound Infection (−)	Total
DM present	12 (38.7%)	19 (61.3%)	31
No DM	6 (9.8%)	55 (90.2%)	61
Total	18 (19.6%)	74 (80.4%)	92

Crude OR 5.79; 95% CI 1.91–17.57; Fisher’s exact test *p* = 0.002.

## Data Availability

The datasets generated and analysed during the current study are available from the corresponding author on reasonable request. Individual patient data are not publicly available due to privacy and ethical restrictions.
